# General Practice-led urgent care practice vs. emergency room – satisfaction of ambulatory patients with low urgency medical problems

**DOI:** 10.1080/13814788.2025.2520218

**Published:** 2025-06-27

**Authors:** Katharina Schmalstieg-Bahr, Bastian Bessert, Penelope-Sophie Peters, Johanna Sophie Bobardt, Ulrich Mayer-Runge, Martin Scherer, Jan Oltrogge-Abiry

**Affiliations:** ^a^ Department of General Practice and Primary Care, University Medical Center Hamburg-Eppendorf, Hamburg, Germany; ^b^ Interdisciplinary Central Emergency Department, University Medical Center Hamburg-Eppendorf, Hamburg, Germany

**Keywords:** Primary care, urgent care practice, ambulatory emergency room patients, waiting time, patient satisfaction

## Abstract

**Background:**

Emergency room (ER) utilisation by ambulatory patients with low urgency medical problems leads to ER-capacity use and long waiting times. Establishing General Practice (GP)-led urgent care practices (UCP) adjacent to ERs allows to triage patients from the ER to the UCP. However, patients may perceive themselves as ER-cases and expect ER-treatment including extensive diagnostics.

**Objectives:**

To assess UCP-patients’ satisfaction compared to ambulatory ER-patients.

**Methods:**

Sub-analysis (11/2019–01/2020) of a prospective, monocentric observational study at the University Medical Centre Hamburg-Eppendorf ER and co-located UCP focusing on patient survey data including demographics, waiting time and diagnoses. Satisfaction, uncertainty and appropriateness of waiting time was assessed with 4-point Likert-scales.

**Results:**

Analysing 1196 UCP- and 597 ER-patients, patient satisfaction correlated positively with perceived appropriate waiting time in both groups. But more UCP-patients deemed their waiting time appropriate (76.7% vs. 70.4%; *p* = 0.004) and reported to be very satisfied with the treatment (64.7% vs. 55.8%; *p* < 0.001). Time until the first physician contact was nearly equal, but the entire length of stay was shorter in the UCP (104 ± 88.0 min vs. 179 ± 301 min; *p* < 0.001). In both groups, satisfaction was reduced by on-going uncertainty after the visit, but uncertainty was higher among UCP-patients (32% vs. 25%; *p* = 0.003). Age, gender or diagnosis had no influence on patients’ satisfaction. More UCP-patients stated that today’s problem could have been treated by a GP (57% vs. 15%; *p* < 0.001) and were advised to follow up in an outpatient setting.

**Conclusions:**

Treating patients in an UCP does not lead to overall dissatisfaction.

## KEY MESSAGES

Emergency room (ER)- and urgent care practice (UCP)-patients’ satisfaction correlated positively with a perceived appropriate waiting time, but more UCP-patients stated that their waiting time was appropriate.

Satisfaction correlated negatively with on-going uncertainty after the visit; uncertainty was higher among UCP-patients.

Age, gender or diagnosis had no influence on patients’ satisfaction.

## Introduction

In many countries hospitals face the problem of overcrowded emergency rooms (ER) which can have an adverse effect on patient outcomes and can lead to an increased mortality among ER-patients as a result of long waiting times, treatment delays or errors [1–3]. Studies suggest that many ambulatory ER-patients with low urgency medical problems could be seen by a general practitioner (GP) or another office-based specialist, especially patients who presented to the ER on their own initiative and without prior contact to a physician or emergency medical services (self-referrals) [1,3–7]. An aspect that might enhance the problem is that in countries, such as Germany, the Czech Republic and Greece [8], there is no gatekeeping system. Patients are not required to have a GP and/or may consult any doctor of any specialty without contacting their GP first. If they do not have an established relationship with a GP or another office-based specialist, often hospitals, respectively, the ER, becomes the primary point of contact to the health care system. Several solutions have been discussed to improve the situation: to broaden the ER-spectrum by integrating GPs into the ER-team [9,10], or the establishment and cooperation with (GP-led) urgent care practices (UCP) in proximity to an ER, sometimes referred to as co-located out-of-hours care walk-in-clinics [11–15]. Although the latter would allow to refer patients from ER to the UCP and reduce patient numbers in the ER, even ambulatory patients with low urgency medical problems may see themselves as an emergency case and expect ER-treatment including extensive diagnostics. A study found that besides convenience triggered by time constraints, health anxiety and the wish to seek multidisciplinary medical care in a highly equipped setting were motives for patients to present to the ER [16]. UCP-treatment with limited diagnostic options might not be well perceived. But this is speculative as the UCP-patient’s perspective has not been well researched. Studies either focused on the objective quality of UCP-care [17], or on patients with low urgency medical problems in the ER [6,16,18,19] or in similar, but other settings, such as nurse practitioner-led UCPs [20] or UCPs that were not adjacent to an ER and took patients by appointment as well [21]. This analysis aims to reduce the gap by focusing on the satisfaction of UCP-patients in the setting of co-location and out-of-hours-care without appointments.

## Methods

### Study design

This is a sub-analysis of a prospective, monocentric pre–post comparative study. The main study focused on the utilisation of a GP-led urgent care practice (UCP) adjacent to an ER in a tertiary care centre by comparing the time period before and after the UCP opened. It demonstrated that the opening lead to a reduction of ER-patients receiving outpatient care, shorter treatment times, and fewer treatment discontinuations in the ER. Furthermore, it showed that most UCP-patients were able to receive definitive care in the UCP and did not need to be referred to the ER [22]. This current sub-analysis uses data from the post-opening period and focuses on the satisfaction of ambulatory patients with low urgency medical problems that were treated in the co-located UCP compared to those who received ER-care. Patients that were hospitalised after being seen in the ER were not included. The main study was conducted at the University Medical Centre Hamburg-Eppendorf, Hamburg, Germany (UKE) from 08/2019 to 09/2019 (pre-period) and from 11/2019 to 01/2020 (post-period) following the opening of the UCP on UKE grounds on the 1 October 2019. For the main study a patient survey conducted in the ER and UCP as well as electronic medical records (EMR) from both study periods were used. This sub-analysis from the post-opening period includes patient survey data from an additional questionnaire that participants were asked to fill out AFTER their treatment.

### Setting: The GP-led urgent care practice

The UCP was located in the same building (25 m distance from the UKE ER), however, independently run by the Association of Statutory Health Insurance Physicians Hamburg (Kassenärztliche Vereinigung Hamburg, KVHH) including its own computer system. All nurses and medical assistants working at the UCP were employed by the KVHH; all UCP-GPs were also UKE primary care physicians. The UCP was open from Monday–Thursday from 6 pm to midnight, Friday 5 pm to midnight and on weekends and national holidays from 8am to midnight. Ambulatory patients could directly present to the UCP or were sent there during UCP-opening hours by ER-triage staff if they presented to the ER and UCP-treatment seemed appropriate. Diagnostic testing in the UCP was limited to vitals, ECG, urine dipstick and three point-of-care rapid tests (CRP, troponin, D-Dimer). Patients that required – after history taking and physical exam – more than these tests urgently and/or hospital admission, needed to be referred to the ER by the UCP-physician. If a non-urgent investigation was appropriate, patients were advised to follow-up with their GP or an office-based specialist.

### Patient population and recruitment

Eligible to participate were patients ≥ 18 years that walked into the UCP or ER with a low urgency medical problem (based on self-reported complaints). Patients needed to be able to give a written consent in German or English. Excluded were minors, patients brought to the ER by emergency services, with significant language barrier and/or patients being unable to consent. Recruitment took place daily between 4 pm and midnight in the ER and during the above-mentioned opening hours in the UCP. Patients that consented to participate received the paper-based survey and were asked to complete after their treatment, but before leaving the UCP/ER. Sociodemographic data was taken from the questionnaire published in the main study [22]. In the additional post-treatment satisfaction questionnaire (Supplementary Data), patients were asked regarding their overall treatment satisfaction, perception of waiting time, on-going uncertainty after the treatment and recommended follow-up. Data regarding the diagnosis was either retrospectively taken from the patients’ EMR (ER-patients) or recorded paper-based the day of the visit by the treating physicians (UCP-patients). Actual waiting time was estimated based on EMR data and patient-reported data regarding the registration time, first physician contact and discharge (Supplementary Data ‘Patient Questionnaire’).

### Statistical analysis

ICD diagnoses were translated to ICPC-2 codes to better summarise single diagnoses into groups.

For the descriptive analysis, responses to the 4-point Likert-scale items were dichotomised, as depicted in the respective tables. For bivariate comparisons of questionnaire items between ER and UCP, unpaired chi-square tests were used for categorical variables, and independent t-tests were applied for continuous variables (age, waiting time and length of the entire stay). To analyse the primary variable ‘treatment satisfaction, ‘we defined two levels of satisfaction: ‘general treatment satisfaction’ (participants who agreed or strongly agreed with the statement ‘I am generally satisfied with my medical care today’) and a subgroup with ‘very high treatment satisfaction’ (participants who only strongly agreed with the above statement. We conducted multivariate logistic regression analyses to estimate the odds ratios for belonging to the group with ‘very high treatment satisfaction’ (Table 3) as well as for belonging to the group with ‘ongoing uncertainty after discharge’ in our samples of ER- and UCP-patients (Supplementary Table 1). Variance inflation factors (VIFs) were calculated for all independent variables, revealing no indication of problematic multicollinearity. Additionally, the assumption of linearity in the logit for the continuous variable age was assessed by including an interaction term, which was not statistically significant in the models. A p-value of < 0.05 was considered statistically significant for all analyses. All statistical analyses were performed using SPSS version 26.

## Ethical approval

The study was approved by the Ethics Committee of the Hamburg Medical Association (No. PV-7035).

## Results

**Figure 1.**
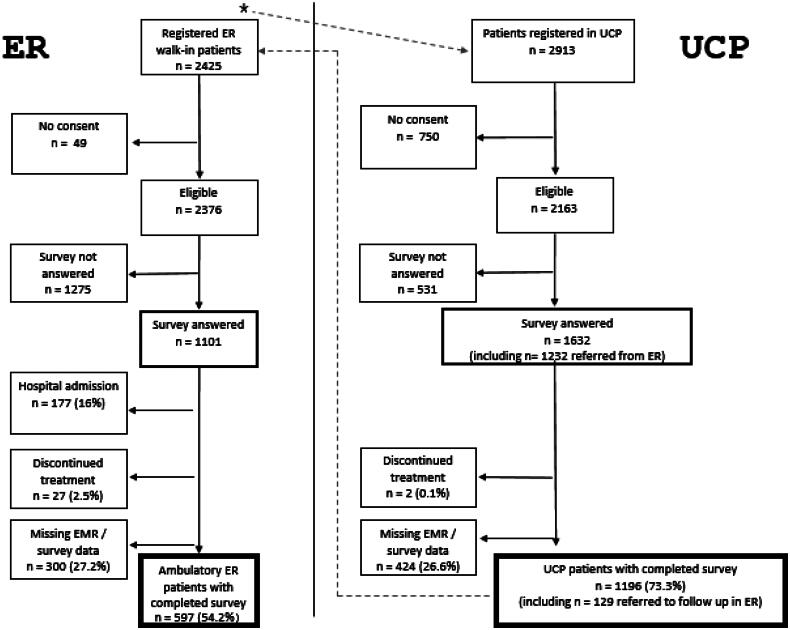
Overview of the recruitment process (November 2019–January 2020). *Depending on their self-reported symptoms, some patients who initially talked to the ER-triage staff were directly sent to the UCP and did not become registered ER-patients. But only those who consented and answered the survey were asked if they came from the ER.

Figure 1 gives an overview of the recruitment process. Depending on their self-reported symptoms, some patients who initially talked to the ER-triage staff were directly referred to the UCP without becoming registered ER-patients. It is unknown how many of these patients that were sent from the ER to the UCP did not consent to participate in the study and/or did not answer the survey. Only those who consented and answered the survey were asked if they came from the ER. Patients that were referred from the UCP to the ER for further testing and/or possible admission were asked to answer the survey before leaving the UCP and check ER as their recommended follow-up. Patients’ characteristics are depicted in Table 1.

**Table 1.** Patients’ characteristics.

	ER (*n* = 597)	UCP (*n* = 1196)	p
Gender	Females 52.6% (*n* = 314) Males 47.4% (*n* = 283)	Females 54.1% (*n* = 647) Males 45.8% (*n* = 548) Non-binary 0.1% (*n* = 1)	*p* = 0.64
Age (years ± SD)	42.0 ± 17.0	40.1 ± 15.7	*p* = 0.003
Diagnostic groups (according to ICPC-2)			
Symptom diagnoses	14.6% (*n* = 87)	19.1% (*n* = 229)	*p* = 0.02
Infections	12.9% (*n* = 77)	36.7% (*n* = 439)	*p* < 0.001
Injuries	42.4% (*n* = 253)	11.8% (*n* = 141)	*p* < 0.001
Other diagnoses	29.8% (*n* = 178)	31.9% (*n* = 382)	*p* = 0.36

ER: Emergency room; UCP: Urgent care practices; SD: Standard deviation.

UCP-patients were younger and had significantly less injury- but more infection-diagnoses. Symptom diagnoses were more common in the UCP, but the difference was not significant. Table 2 depicts the results of the participants’ assessment after their treatment in comparison to their waiting times and lengths of stay. The waiting time to first physician contact showed no significant difference between ER und UCP with 70.4% of ER- and 76.7% of UCP-patients who found their waiting time appropriate. Although there was a difference regarding the entire length of stay (ER: 178.5 min vs. UCP: 104.2 min; *p* < 0.001).

**Table 2.** Comparison of ER- and UCP-patients regarding satisfaction, waiting time and on-going uncertainty after the consultation.

	ER (*n* = 597)	UCP (*n* = 1196)	p
Mean waiting time to physician contact (±SD)	82.6 min ±96.0*	91.5 min ± 97.0**	*p* = 0.07
Mean length of entire stay (±SD)	178.5 min ± 300.6***	104.2 min ± 87.6****	*p* < 0.001
*My waiting time was appropriate.* (‘agreed’ or ‘strongly agreed’)	70.4% (*n* = 420)	76.7% (*n* = 917)	*p* = 0.004
*There is still an uncertainty about my health condition.* (‘agreed’ or ‘strongly agreed’) Defined as: ‘ongoing uncertainty after discharge’	25.0% (*n* = 149)	31.9% (*n* = 382)	*p* = 0.003
*I am generally satisfied with my medical care today.* (‘agreed’ or ‘strongly agreed’)	94.3% (*n* = 563)	96.2% (*n* = 1154)	*p* = 0.035
*I am generally satisfied with my medical care today.* (only ‘strongly agreed’) Defined as: ‘very high treatment satisfaction’	55.8% (*n* = 333)	64.7% (*n* = 774)	*p* < 0.001

Due to missing data, the time to first physician contact and length of stay was calculated based on the following numbers: *n = 588, ** n = 1161, ***n = 588, ****n = 1113. ER: Emergency room; UCP: Urgent care practices; SD: Standard deviation.

‘General treatment satisfaction’ was high in both settings (ER: 94.3% vs. UCP: 96.2%; *p* = 0.035), but the subgroup analysis of ‘very high treatment satisfaction’ showed a more pronounced difference between ER und UCP (ER: 55.8% vs. UCP: 67.7%; *p* < 0.001).

To evaluate factors that influenced patient satisfaction in our sample, we calculated adjusted Odds ratios with ‘very high treatment satisfaction’ as a dependent variable. We found that participants with ‘ongoing uncertainty’ had a lower likelihood of treatment satisfaction in both settings [ER OR:0.37; 95%CI: (0.23–0.59) vs. UCP: OR: 0.42; 95%CI: 0.31–0.55)]. An ‘appropriate waiting time’ was associated with a higher likelihood of treatment satisfaction [ER OR:7.28; 95%CI: (4.74–11.17) vs. UCP: OR:4.10; 95%CI: (3.07–5.48)]. Notably, the discharge diagnosis, the recommended follow-up treatment as well as gender and age showed no association with treatment satisfaction in either setting (Table 3).

**Table 3.** Comparison of multivariable logistic regressions for ED patients versus UCP patients with odds ratios of a ‘very high treatment satisfaction*.

	ER (*n* = 597)		UCP (*n* = 1196)	
	*p*	OR (95%CI)	*p*	OR (95%CI)
Age (per 1 year)	0.184	1.01 (1.00–1.02)	0.466	1.00 (0.99–1.01)
Male gender	0.561	1.12 (0.77–1.63)	0.280	1.15 (0.89–1.49)
**Given ICPC-2 discharge diagnoses**				
Symptom diagnosis	NA	1 [Reference]	NA	1 [Reference]
Infections	0.417	1.35 (0.66–2.76)	0.770	0.95 (0.66–1.37)
Injuries	0.056	1.78 (0.97–3.20)	0.679	0.91 (0.56–1.46)
Other diagnoses	0.191	1.50 (0.82–2.77)	0.442	0.87 (0.60–1.25)
**Reported uncertainty and appropriateness of waiting time**				
’There is still an uncertainty about my health condition.’ (‘agreed’ or ‘strongly agreed’)	<0.001	0.37 (0.23–0.59)	<0.001	0.42 (0.31–0.55)
*My waiting time was appropriate.* *(‘agreed’ or ‘strongly agreed’)*	<0.001	7.28 (4.74–11.17)	<0.001	4.10 (3.07–5.48)
**Participants´ assessment of appropriate healthcare provider**				
*My medical problem could have been treated by my General Practitioner.* (‘agreed’ or ‘strongly agreed’)	0.171	0.67 (0.38–1.19)	0.981	1.00 (0.70–1.43)
*My medical problem could have been treated by a specialist.* (‘agreed’ or ‘strongly agreed’)	0.655	0.91 (0.60–1.38)	0.610	0.91 (0.63–1.32)
**Recommended follow-up**				
No further treatment necessary	NA	1 [Reference]	NA	1 [Reference]
Follow up at ER	0.073	0.50 (0.24–1.07)	0.825	0.95 (0.59–1.53)
Elective hospital admission	0.292	1.86 (0.59–5.84)	0.839	0.89 (0.28–2.79)
Follow up at outpatient department	0.308	0.70 (0.35–1.39)	0.276	0.65 (0.30–1.41)
Follow up with specialist	0.845	1.05 (0.63–1.75)	0.958	0.99 (0.68–1.44)
Follow up with General Practitioner	0.652	0.88 (0.50–1.55)	0.449	1.14 (0.82–1.58)

*‘Very high treatment satisfaction’ was defined as the group of participants who only strongly agreed with the statement ‘I am generally satisfied with my medical care today’ (*n* = 333 E- patients; *n* = 774 UCP-patients).

ER: Emergency room; UCP: Urgent care practices; OR: Odds ratio; CI: Confidence interval; ICPC-2-2: International Classification of Primary Care 2nd edition.

When using patient’s uncertainty as a dependent variable, on-going uncertainty did not show a significant association with the perceived appropriateness of waiting time in both settings. Receiving a specific disease diagnosis – not a symptom diagnosis – reduced the likelihood of on-going uncertainty among UCP-patients, and among ER-patients in two of three ICPC diagnosis groups (Supplementary Table 1).

After the treatment, more UCP-patients were convinced that their problem could have been treated in an outpatient setting, either by a GP or another office-based specialist and were also advised to follow-up in an outpatient setting (Table 4).

**Table 4.** Comparison of ER- and UCP-patients regarding participants’ estimation of the appropriate healthcare provider and the recommended follow-up.

	ER (*n* = 597)	UCP (*n* = 1196)	p
**Participants’ assessment of appropriate healthcare provider**			
My medical problem could have been treated by my General Practitioner (‘agreed’ or ‘strongly agreed’)	15.4% (*n* = 92)	57.2% (*n* = 684)	*p* < 0.001
My medical problem could have been treated by a specialist (‘agreed’ or ‘strongly agreed’)	45.7% (*n* = 273)	68.1% (*n* = 815)	*p* < 0.001
Recommended follow-up*			
No further treatment necessary	25.3% (*n* = 151)	24.5% (*n* = 293)	*p* = 0.714
Elective hospital admission	3.7% (*n* = 22)	1.5% (*n* = 18)	*p* < 0.003
Follow up at ER	8.0% (*n* = 48)	10.8% (*n* = 129)	*p* = 0.066
Follow up at UKE outpatient department	12.7% (*n* = 76)	3.0% (*n* = 36)	*p* < 0.001
Follow up with General Practitioner	22.6% (*n* = 135)	39.0% (*n* = 466)	*p* < 0.001
Follow up with specialist	31.5% (*n* = 188)	23.7% (*n* = 283)	*p* < 0.001

*Some patients (ER: n = 18; UCP n = 22) reported more than one follow up which resulted in 620 answers from 597 ER-patients and 1225 answers from 1196 UCP-patients. ER: Emergency room; UCP: Urgent care practices; UKE: University Hospital Hamburg-Eppendorf.

## Discussion

### Main findings

In both patient groups (ER and UCP), patient satisfaction correlated positively with a perceived appropriate waiting time. But more UCP-patients agreed with the statement that their waiting time was appropriate. The entire length of stay was indeed shorter in the UCP than in the ER. More UCP-patients stated that they were very satisfied with today’s visit. Satisfaction was negatively associated to on-going uncertainty after the visit. This was higher among UCP-patients. Age, gender or the discharge diagnosis had no influence on patients’ satisfaction.

More UCP-patients were advised to follow-up in an outpatient setting and agreed or strongly agreed with the statement that today’s problem could have been treated by a GP or an office-based specialist. However, 45% of ER-patients also agreed or strongly agreed with the later statement. Since patients answered the survey after their ER-/UCP-treatment, it is likely that patients came to that conclusion in hindsight knowing what kind of service/testing they received today.

### Comparison with literature

In line with this study, other studies have demonstrated that UCP-patients were more satisfied than ER-patients [21,23]. Arain et al. [23] included data from two UCPs; one was independent, one was built in a community health centre alongside a number of other primary care services. But neither was adjacent to an ER and data from ER-patients was not considered. Whereas, Chalder et al. [24] included patients from eight ERs with adjacent UCPs as well as from eight ‘traditional’ ERs. UCP-patients were just as satisfied with their overall care as their counterparts in the co-located ER, but reported greater satisfaction in some aspects, such as visit duration, facility cleanliness, time given to discuss healthcare problems, involvement in decision-making, discussion of fears and anxieties and privacy during the consultation [24], aspects that – except visit duration/overall length of stay – were not covered in this study. The latter study also raised an important issue: frequently patients were unable to distinguish between being treated in the ER or the adjacent UCP, which makes any kind of comparison less robust.

But available evidence is inconsistent. A study that mailed a questionnaire to a random sample of patients from 36 GP practices to assess whether they had a recent urgent health problem, where they sought care and how satisfied they were, showed the highest satisfaction rates for patients that received care from their own GP followed by ER-patients and patients who called a health advisory service. UCP-patients reported the lowest satisfaction rates [25].

Another study found no overall significant differences in satisfaction between patients with medically unexplained symptoms and patients whose symptoms were explained by an specific (organic) diagnosis [26]. This is similar to the present results: the type of diagnosis influenced uncertainty, but not satisfaction. Jackson et al. [26] also included satisfaction aspects that were not specifically covered in this study such as satisfaction with information, with style of doctor–patient interaction, with clinic environment, and with patient’s health.

In this analysis, on-going uncertainty in both groups (32% UCP vs. 25% ER) was higher than in a study which described a 10% uncertainty rate among patients in the ER and urgent care setting. The difference could probably be explained by the methods used. DeGennaro et al. sent a survey ‘to a nationally representative panel’ resulting in a longer time period between the treatment and assessment, so on-going uncertainty could have been reduced in the meantime e.g. by having doctor’s appointment [27].

In line with the current results that more UCP-patients, but also a significant percentage of ER-patients were convinced that their medical problem could have been treated by a GP, a study assessing the patient’s view on the co-location of UCPs and ERs found that even the patients who attend the ER had confidence in the skills and competences of the GP [28]. According to the authors patients thought that for particular problems, e.g., wounds, ER-treatment was more appropriate despite having general confidence in their GP.

### Strengths and limitations

To the best of our knowledge, our study was the first investigating the introduction of a UCP co-located to an ER at a university hospital in Germany. Due to the daily recruitment and case numbers, one can assume that our sample is representative for the investigated care setting. However, there may be a selection bias since no data was captured from patients who discontinued the treatment. Organisational adjustments such as boosting staff levels were not made during the study period, but seasonal factors (e.g., more patients with infections during winter) cannot completely be excluded. Since this sub-analysis is mainly based on self-reported data, subjectivity was unavoidable. It is unknown why some patients initially talked to the ER-triage staff instead of directly presenting to the UCP. Possible reasons may include that they were unaware of the just opened UCP. But the survey did not specifically assess that. Another limitation is that we used a subgroup defined as ‘very high treatment satisfaction’ as the dependent variable in our regression analysis, since our initial operationalisation of ‘general treatment satisfaction’ was reported by >94% of participants, limiting its applicability for identifying associations. Furthermore, the two open-ended questions (Supplementary Data ‘Patient Questionnaire’) were not included in this analysis and may provide a better understanding to why participants agreed with the statement that their medical problem could have been treated in an outpatient setting. Reason may include that they presented during the weekend when outpatient facilities were closed. After all, it was a monocentric study. Results might differ at a non-maximum care hospital.

## Conclusion and implications for practice and research

Treating patients with low urgency medical problems in a GP-led UCPs instead of an ER does not seem to cause patient’s dissatisfaction in regards to the aspects of treatment satisfaction and perceived appropriateness of waiting time. On the contrary: UCP-patients reported higher satisfaction rates although they received no or only limited diagnostic testing. But uncertainty after the visit was higher among UCP-patients. From that point of view it seems promising to establish GP-led UCPs adjacent to ERs, although others aspects not covered in this study need to taken into account. The qualitative analysis of the open-ended survey questions will provide additional insight to the issue raised above and further qualitative studies may give a more profound view of other dimensions that could influence the patient’s satisfaction, e.g. patient–physician-relationship.

## Supplementary Material

Supplemental Material
