# Supplementary material for: General Practice-led urgent care practice vs. emergency room – satisfaction of ambulatory patients with low urgency medical problems
Source: Eur J Gen Pract. 2025 Jun 27;31(1):2520218. doi: 10.1080/13814788.2025.2520218 (PMC12207775; doi:10.1080/13814788.2025.2520218)
Supplement: Supplemental Material [file IGEN_A_2520218_SM5949.zip › suppl_data/ejgp-2025-0039-File003.docx]

Supplementary Table 1: Comparison of multivariate logistic regressions for ER-patients versus UCP-patients with odds ratios of an ‘ongoing uncertainty after discharge’^*^

|  | **ER**  **(n=****597)** | | **UCP**  **(n=****1196)** | |
| --- | --- | --- | --- | --- |
|  |  |  |  |  |
|  | **p** | **OR (95%CI)** | **p** | **OR (95%CI)** |
| Age (per 1 year) | 0.596 | 1.00 (0.98–1.01) | 0.825 | 1.00 (0.99–1.01) |
| Male gender | 0.517 | 0.87 (0.57–1.32) | 0.789 | 1.04 (0.79–1.36) |
|  |  |  |  |  |
| **Given ICPC-2 discharge diagnoses** |  |  |  |  |
| Symptom diagnosis | NA | 1 [Reference] | NA | 1 [Reference] |
| Infections | 0.052 | 0.50 (0.25–1.01) | **<0.001** | 0.40 (0.28–0.58) |
| Injuries | **<0.001** | 0.20 (0.11–0.37) | **<0.001** | 0.32 (0.19–0.52) |
| Other diagnoses | **0.010** | 0.46 (0.26–0.84) | **<0.001** | 0.53 (0.37–0.76) |
|  |  |  |  |  |
| **Reported satisfaction and appropriateness of waiting time** |  |  |  |  |
| *I am generally satisfied with my medical care today.*  *(only “strongly agreed”)* | **<0.001** | 0.37 (0.33–0.59) | **<0.001** | 0.42 (0.31–0.55) |
| *My waiting time was appropriate.*  *(“agreed” or “strongly agreed**” )* | 0.131 | 0.70 (0.44–1.11) | 0.332 | 1.18 (0.85–1.64) |
|  |  |  |  |  |
| **Recommended follow-up** |  |  |  |  |
| No further treatment necessary | NA | 1 [Reference] | NA | 1 [Reference] |
| Follow up at ER | **0.001** | 3.81 (1.68–8.63) | **<0.001** | 8.28 (5.06–13.56) |
| Elective hospital admission | **<0.001** | 7.81 (2.69–22.70) | **<0.001** | 8.93 (2.96–26.98) |
| Follow up at outpatient department | **<0.001** | 5.10 (2.41–10.78) | **0.001** | 3.67 (1.68–8.05) |
| Follow up with specialist | **<0.001** | 3.39 (1.82–6.30) | **<0.001** | 4.37 (2.94–6.48) |
| Follow up with General Practitioner | **0.010** | 2.49 (1.24–4.99) | **<0.001** | 1.89 (1.30–2.75) |
|  |  |  |  |  |
| **Participants´ assessment of appropriate healthcare provider** |  |  |  |  |
| My medical problem could have been treated by my General Practitioner  (“agreed” or “strongly agreed”) | 0.907 | 1.04 (0.54–2.02) | 0.239 | 1.25 (0.86–1.80) |
| My medical problem could have been treated by a specialist  (“agreed” or “strongly agreed”) | 0.700 | 0.91 (0.58–1.45) | 0.853 | 1.04 (0.71–1.52) |

^*^“ongoing uncertainty after discharge” was defined as the group of participants that agreed or strongly agreed with the statement: “There is still an uncertainty about my health condition”.

ER: emergency room, UCP: urgent care practices, OR: odds ratio, CI: confidence interval, ICPC-2-2: International Classification of Primary Care 2nd edition
