# Supplementary material for: General Practice-led urgent care practice vs. emergency room – satisfaction of ambulatory patients with low urgency medical problems
Source: Eur J Gen Pract. 2025 Jun 27;31(1):2520218. doi: 10.1080/13814788.2025.2520218 (PMC12207775; doi:10.1080/13814788.2025.2520218)
Supplement: Supplemental Material [file IGEN_A_2520218_SM5949.zip › suppl_data/ejgp-2025-0039-File004.docx]

##### Patient questionnaire for the study:

***Care episodes of emergency department patients***

**We kindly ask you to evaluate your today´s treatment in our emergency department. Please indicate how much you agree with the following statements.**

|  | **Strongly disagree** | **Disagree** | **Agree** | **Strongly agree** |
| --- | --- | --- | --- | --- |
| **1 I am generally satisfied with my medical care today.** | ⬜ | ⬜ | ⬜ | ⬜ |
| **2 My waiting time was appropriate.** | ⬜ | ⬜ | ⬜ | ⬜ |
| **3 There is still an uncertainty about my health condition.** | ⬜ | ⬜ | ⬜ | ⬜ |
| **3 My medical problem could have been managed by a general practitioner as well.** | ⬜ | ⬜ | ⬜ | ⬜ |
| **4 My medical problem could have been managed by a resident specialist as well.** | ⬜ | ⬜ | ⬜ | ⬜ |

| **5 What kind of follow-up procedure was recommended to you?** (please check) | |
| --- | --- |
| **No further treatment necessary** ⬜ | **Appointment for a hospital admission**⬜ |
| **Follow-up appointment at our emergency department** ⬜ | **Follow-up appointment at outpatient department of a hospital** ⬜ |
| **Further treatment by your family doctor** ⬜ | **Further treatment by a specialist** ⬜ |
| Other follow-up treatment (please enter)_____________________________________________ ⬜ | |

| **6 Please indicate your waiting time until the beginning of your treatment today.**  (in hours and/or minutes) | ___________ (hours)  ___________ (minutes) |
| --- | --- |
| **7 Please indicate how much time you have spent in total in this emergency department today.**  (in hours and/or minutes) | ___________ (hours)  ___________ (minutes) |

| **Why didn’t you attend to a general practitioner or no another doctor´s office due to your current medical problem? (Please only a few keywords)** |
| --- |

| **Is there anything else you want to tell us?** |
| --- |

🌸
